# The sperm mitochondria: clues and challenges

**DOI:** 10.1590/1984-3143-AR2022-0131

**Published:** 2023-02-13

**Authors:** Diego Bucci, Marcella Spinaci, Ivan Cunha Bustamante-Filho, Salvatore Nesci

**Affiliations:** 1 Department of Veterinay Medical Sciences, University of Bologna, Bologna, Italy; 2 Universidade do Vale do Taquari, Lajeado, RS, Brasil

**Keywords:** sperm metabolism, bioenergetics, hexose uptake

## Abstract

Sperm cells rely on different substrates to fulfil thei energy demand for different functions and diverse moments of their life. Species specific mechanism involve both energy substrate transport and their utilization: hexose transporters, a protein family of facilitative passive transporters of glucose and other hexose, have been identified in spermatozoa of different species and, within the species, their localization has been identified and, in some cases, linked to specific glycilitic enzyme presence. The catabolism of hexose sources for energy purposes has been studied in various species, and recent advances has been made in the knowledge of metabolic strategies of sperm cells. In particular, the importance of aerobic metabolism has been defined and described in horse, boar and even mouse spermatozoa; bull sperm cells demonstrate to have a good adaptability and capacity to switch between glycolysis and oxidative phosphorylation; finally, dog sperm cells have been demonstrated to have a great plasticity in energy metabolism management, being also able to activate the anabolic pathway of glycogen syntesis.

In conclusion, the study of energy management and mitochondrial function in spermatozoa of different specie furnishes important base knowledge to define new media for preservation as well as newbases for reproductive biotechnologies.

## Introduction

Spermatozoa are highly specialized cells that are aimed at delivering the male DNA into the new generation subjects. To do so, after being produced by the testicle, matured and stored in the epididymis, they are released in the female genital tract, where they begin the long journey to the female gamete in order to reach it and act for the fertilization process.

In mammalian sperm, between ejaculation and fertilization, there can also be another important step in sperm life, that is not occurring in natural breeding animals, but usually occurs when artificial reproduction techniques are used: conservation.

Irrespective of the imminent fate of sperm cells, it should be stated that they need some energy substrate to adapt to the environment, maintaining homeostasis and movement.

This review aims at pointing out the most recent knowledge on sperm metabolism in terms of fuelling supply, utilization of substrates and metabolic strategies, and balance between anaerobic and aerobic pathways.

Most of the knowledge on different mammalian species has been presented and reviewed by outstanding research groups and colleagues during the last years (Boguenet et al., 2021; Moraes and Meyers, 2018; Peña et al., 2022; Rodriguez-Gil, 2006; Rodríguez-Gil and Bonet, 2016; Varner et al., 2015) and we also focused on some specific aspects of sperm metabolism (Bucci et al., 2011). We therefore invite the reader to refer also to those review papers to widen the knowledge on the theme. In this review, we will also present some unpublished data from our labs regarding the balance and equilibrium between anaerobic and oxidative metabolism.

## Energy sources for sperm cells. Not only sugar

An interesting paper by Storey (2008) focused on the regulatory and fuelling role of sugars in mammalian sperm life and activity (Storey, 2008). The author thoroughly revised a large number of studies dating back to the 1940s, in which it was first studied and reported how important and precious sugar fuelling was for sperm function.

Of particular interest, among the first studies on sperm metabolism, were the researches reported in Storey’s review by Lardy and Philips and co-workers, as well as the impressive work published by Mann (for reviewing and references see (Storey, 2008)); those first experimental work were aimed at defining the role of fuelling sugars for maintaining the most evident sperm function, motility, as well as to maintain sperm fertilizing ability. From that point on, a large interest was given to sugars and their role in sperm preservation under liquid storage conditions. It is well known that different species may rely on hexoses for their metabolism (Fernández-Novell et al., 2004; Medrano et al., 2005; Peña et al., 2022). Nevetheless, other mechanisms of fuelling have been demonstrated over time (Brooks and Mann, 1972, 1973; Hutson et al., 1977; Medrano et al., 2006a) in different species, thus demonstrating that the direct impact of mitochondrial metabolization of energy sources could play a major role in energy obtainment from different species.

The uptake of hexose monosaccharides is exerted by different members of a protein family of carriers and these comprises the so called GLUTs (glucose transporters) which have different specificities for the substrates hexoses (Bucci et al., 2011): CLASS I transporters, (GLUT 1, 2, 3, 4, and 14) are mainly glucose transporters (excepting for GLUT2, that transport also fructose); CLASS II transporters (Glut 5, 7, 9, 11), fructose or double affinity transporters; CLASS III transporters (GLUT 6, 8, 10, 12 and HMIT), with hight affinity for glucose and a different structure if compared with CLASS I and II ones.

These proteins have been studied in sperm from different species (Angulo et al., 1998; Bucci et al., 2010a, b, 2011; Sung and Moley, 2007) such as human, rat, and bull sperm cells. GLUTs 1, 2, 3, 4, and 5 (Angulo et al., 1998) show species specific localization within sperm head and tail, and each GLUT shows a different distribution within the same species. The immunocytochemistry results were also validated with Western Blot analysis. Our group studied GLUT 1, 2, 3, 5 in horse, donkey, boar and dog sperm cells (Bucci et al., 2010a), defining, with the same experimental design as Angulo, the presence and abundance of glucose transporters (immunocytochemistry and western blotting). Finally, GLUT 8 and 9 were studied in mouse testis and mature spermatozoa (Sung and Moley, 2007)

Interesting studies have regarded the relationship between GLUTs activity and the metabolism of sperm cells: in the early 2000s, Rigau and colleagues showed that metabolic plasticity of dog spermatozoa could be related to GLUTs localization (Fernández-Novell et al., 2004; Rigau et al., 2001, 2002); similarly, it was demonstrated that GLUT 3 co-localizes with Hexokinase I in pig spermatozoa(Medrano et al., 2006b), and this could strictly link the activity of the transporter and the enzymes responsible for the metabolization of the transported substance.

Finally, we demonstrated that GLUT 3 and 5 in dog spermatozoa (Bucci et al., 2010a) undergo relocalization after incubation under capacitating conditions; again, a swift in metabolic rate of the sperm cells induces a modification of the localization of the suppliers of energy substrates.

Monocarboxilate transporters (MCTs) have been recently described in spermatozoa, specifically MCT1 has been identified in the sperm head; these transporters are responsible for transport of pyruvate/lactate and their presence could play a promising role in the production of next-generation sperm preservation extenders (Peña et al., 2022).

## Different strategies for energy obtainment and mitochondria

Since a long time, it was recognised that spermatozoa from different species have different metabolic strategies to obtain energy for their metabolic activity (Bucci et al., 2011; Gibb and Aitken, 2016; Peña et al., 2022; Rodriguez-Gil, 2006; Rodríguez-Gil and Bonet, 2016; Varner et al., 2015); this section will furnish a brief description of the different energy obtainment strategies exerted by different species to sustain sperm function.

**Figure 1:**
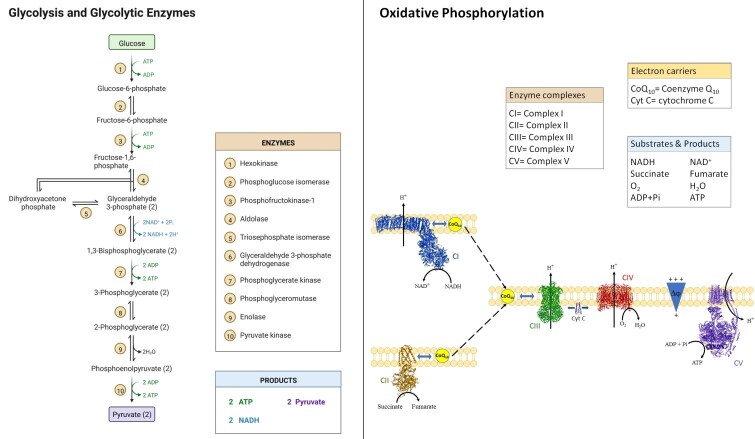
Representation of the anaerobic and aerobic energy obtainment pathways.

It is noteworthy to point out that sperm cells may use preferably the anaerobic pathway (glycolysis) or the aerobic one (oxidative phosphorylation) to obtain energy (see Figure 1).

### Boar spermatozoa

Boar sperm cells are recognized as a typical phenotype of slow, short-living cells (Marin et al., 2003; Rodriguez-Gil, 2006); this fact is related to the physiology of reproduction in this species, in which the female, thus having a long lasting estrous (2-4 days) has a receptibility peak in the middle 24 hours of the estrous period; it is reported that sperm cells in the female genital tract could live no more than 18 hours (Johnson et al., 2000).

Studies on metabolism of boar sperm cells defined these cells as primarily glycolytic, showing up to 95% of anaerobic metabolism, as revealed by mass spectrometry studies (Marin et al., 2003). In addition, the presence of hexokinase and the metabolizing rate of glucose have led the researcher to sustain this dogma (Fernández-Novell et al., 2004; Medrano et al., 2005, 2006b). Anyway, the intervention of mitochondria in boar sperm cells metabolism cannot be discarded, as different studies have shown the presence of an active metabolism of mitochondrial substrates (Brooks and Mann, 1973; Medrano et al., 2006a); in addition, a recent study from our laboratories, carried out using different specific inhibitors of the electron transfer chain, demonstrated that boar spermatozoa have an active mitochondrial metabolism and that mitochondria preferably rely on complex I instead of complex II to oxidize substrates (Nesci et al., 2020). This finding is opening a new interest on boar sperm mitochondria and their actual role in energy supply.

### Dog spermatozoa

Dog spermatozoa are retained as the opposite phenotype of boar sperm: they are fast cells, with a great surviving capacity in the female genital tract (more than 10 days). Again, this situation mirrors the reproductive physiology demands of this species, in which the bitch has a long lasting estrous (till 9 days) and sexual receptivity is not always synchronous with ovulation (up to 11 days from ejaculation to fertilization) (Foutouhi and Meyers, 2022). Therefore, sperm cells from the dog must have the possibility to remain alive and functional for longer time, and evolute different metabolic strategies: high capacity to metabolize glucose, (Fernández-Novell et al., 2004), metabolic plasticity (Bucci et al., 2010a; Rigau et al., 2001, 2002), ability to activate anabolic glycogen synthesis pathways (Ballester et al., 2000) and to further use glycogen for highly demanding functions (Albarracín et al., 2004). Meyers and colleagues (Foutouhi and Meyers, 2022) report that canine spermatozoa demonstrated to have a high capacity to increase their oxidative metabolism when properly treated and that, in case of disruption of oxidative metabolism, they can in a certain way supply with hexose utilization.

### Horse spermatozoa

Horse spermatozoa are mostly oxidative cells: it is reported that their metabolic strategy could be defined as “live fast, dye young”. Several researches from the group of Fernando Peña (Davila et al., 2015, 2016; González-Fernández et al., 2009; Ortiz-Rodriguez et al., 2021; Peña et al., 2022, 2015; Plaza Dávila et al., 2015) have clearly demonstrated that active and fit mitochondria are needed for horse sperm functioning in the proper way; in particular, the approach chosen in these studies, (recently applied also in our labs to better delineate the relationship between sperm motility, ROS production and mitochondrial function (Giaretta et al., 2022)) was selective inhibition of electron transfer chain (ETC) of respiratory complexes to determine their role in ETC function and ROS production. Other studies, by Meyers and colleagues ( Darr et al., 2016a , b; Foutouhi and Meyers, 2022; Meyers et al., 2019; Moraes and Meyers, 2018) deepened the study of the role of mitochondria in stallion semen function, using different techniques to determine Oxygen Consumption Rate (OCR) and ATP production. Finally, the group of Zamira Gibb furnished some very interesting clues on mitochondria potentiality (Gibb et al., 2014, 2015; Gibb and Aitken, 2016; Swegen et al., 2016; Varner et al., 2015). These intense studies have demonstrated that an impairment of the mitochondrial function is strongly deleterious for horse sperm function and that the key point to support a good functionality and survival of horse sperm cells is the presence of intact mitochondria.

### Bull spermatozoa

Bull spermatozoa have been the first ones to be studied, as reported in the milestone review by Storey (Storey, 2008). After a relatively long period in which sperm metabolism was not the center of the studies in bull semen, new interest was focused on these features. (Bulkeley et al., 2021; Chatterjee et al., 2001; Contri et al., 2010; Moraes et al., 2021; Thys et al., 2009). Bull sperm cells are probably the most widely used in AI techniques all over the world and, generally speaking, they are easily cryopreserved; as a consequence, we register a great advance in the application of AI techniques in spite of basic research on metabolic features.

Bull spermatozoa can rely both on glycolysis and oxidative phosphorylation pathways: in normal conditions the two pathways play an integrated role as expected from somatic cells metabolism (Vishwanath and Shannon, 2000). In these conditions, mitochondria are “coupled” and their functionality guarantees a good function of the metabolic machinery (Bulkeley et al., 2021; Moraes et al., 2021) and sustains motility. Anyway, after cryopreservation, bull sperm mitochondria do not work properly, probably because of cryo-injuries, as we demonstrated in recent research from our labs (Algieri et al., 2022), in which we showed that bull frozen sperm mitochondria are uncoupled, as mitochondrial respiration does not support the ATP synthesis, in contrast with what was observed by other Authors in freshly ejaculated semen (Bulkeley et al., 2021). In another research (under review) we studied the action of different ETC inhibitors on bull frozen sperm cells. The results clearly showed that these cells are more resistant than horse ones to ETC inhibition, and that only inhibition of complex III is able to significantly decrease mitochondrial membrane potential and motility (as observed also in fresh semen) (Bulkeley et al., 2021). These findings together seem to contrast with the fact that frozen semen from bull have overall a good fertilizing ability; we believe that in case of oxidative phosphorylation breakdown, the glycolytic pathway is able to sustain motility and sperm cell homeostasis.

### Mouse spermatozoa

Mouse sperm cells were studied under different aspects, one of these was sperm metabolism, and they were considered for a long time strictly glycolytic cells (Ford, 2006; Krisfalusi et al., 2006; Mukai and Okuno, 2004), as motility is strictly related to an active glycolytic pathway within the cell. After almost a decade from these studies, some insights were focused also in mouse sperm mitochondrial activity (Tourmente et al., 2015), thus demonstrating that mitochondria may have a role in energy production; a recent work by the same Authors (Tourmente et al., 2022), applying novel techniques already used in bull, boar and canine spermatozoa(Foutouhi and Meyers, 2022), demonstrated that mitochondrial ATP production plays a pivotal role in capacitation process in mouse spermatozoa, which shift their metabolism from a highly glycolytic one toward an oxidative one. As a technical note, perhaps some investigation on possible differences in sperm metabolism of the most used mouse strains could reveal the best model for translational reseach.

## Concluding remarks and future perspectives

The study of sperm metabolism has undergone different moments of interest by the scientific community; anyway, this brief review underlines that knowledge on this topic is not only essential, but can represent an interesting research field. New methodologies and instruments have been developed in the last ten years, thus permitting to shift the approach toward more sensible analysis with respect to those available in the past decades. This led to new discoveries and to update some dogma that seemed to be, as per dogma definition, untouchable. Instead, the role of sperm cells mitochondria has grown in importance and the possibility to study more deeply these organelles showed that they have different roles in different species, and could show a really surprising metabolic plasticity that could be well exploited to develop new preservation strategies or to permit a better control of *in vitro* sperm activation (capacitation and acrosome reaction). Only in mouse sperm mitochondrial metabolism was studied under capacitating conditions (Tourmente et al., 2022), but this approach is becoming really precious also to control capacitation in species in which in vitro fertilization IVF works well, such as bovine and porcine, and also in species in which only recently IVF protocols have been described and actuated (Felix et al., 2022).

The knowledge of basic sperm metabolism of each species, and the possibility to study at individual level the metabolic features will be of absolute interest in the future for the formulation of new extenders; current research is looking for new preservation strategies (Gibb et al., 2015; Rizkallah et al., 2022) possibly avoiding the need to cool semen to too low temperature. This technique could have a great impact on sperm preservation and business, but new extenders should be formulated in order to get the best results in terms of sperm survival, bacterial growth control and fertility.
